# Neurogenic mediators contribute to local edema induced by *Micrurus lemniscatus* venom

**DOI:** 10.1371/journal.pntd.0005874

**Published:** 2017-11-21

**Authors:** Luciana Lyra Casais-e-Silva, Catarina Teixeira

**Affiliations:** 1 Laboratory of Neuroimmunoendocrinology and Toxinology, Department of Bioregulation, Institute of Health Sciences (ICS), Federal University of Bahia, Salvador, Bahia, Brazil; 2 Laboratory of Pharmacology, Butantan Institute, Sao Paulo, SP, Brazil; Monash University, AUSTRALIA

## Abstract

**Background/Aims:**

*Micrurus* is one of the four snake genera of medical importance in Brazil. Coral snakes have a broad geographic distribution from the southern United States to Argentina. Micrurine envenomation is characterized by neurotoxic symptoms leading to dyspnea and death. Moreover, various local manifestations, including edema formation, have been described in patients bitten by different species of *Micrurus*. Thus, we investigated the ability of *Micrurus lemniscatus* venom (MLV) to induce local edema. We also explored mechanisms underlying this effect, focusing on participation of neuropeptides and mast cells.

**Methodology/Principal findings:**

Intraplantar injection of MLV (1–10 μg/paw) in rats caused dose- and time-dependent edema with a peak between 15 min and 1 h after injection. MLV also induced degranulation of peritoneal mast cells (MCs). MC depletion by compound 48/80 markedly reduced MLV-induced edema. Pre-treatment (30 min) of rats with either promethazine a histamine H_1_ receptor antagonist or methysergide, a nonselective 5-HT receptor antagonist, reduced MLV-induced edema. However, neither thioperamide, a histamine H_3_/H_4_ receptor antagonist, nor co-injection of MLV with HOE-140, a BK_2_ receptor antagonist, altered the response. Depletion of neuropeptides by capsaicin or treatment of animals with NK_1_- and NK_2_-receptor antagonists (SR 140333 and SR 48968, respectively) markedly reduced MLV-induced edema.

**Conclusions/Significance:**

In conclusion, MLV induces paw edema in rats by mechanisms involving activation of mast cells and substance P-releasing sensory C-fibers. Tachykinins NKA and NKB, histamine, and serotonin are major mediators of the MLV-induced edematogenic response. Targeting mast cell- and sensory C-fiber-derived mediators should be considered as potential therapeutic approaches to interrupt development of local edema induced by *Micrurus* venoms.

## Author summary

*Micrurus* venoms have neurotoxic activity that is responsible for the serious sequelae in human envenomation. However, various local manifestations of envenoming have been described in patients bitten by different *Micrurus* species and edematogenic activity has been experimentally demonstrated. Despite the low frequency of edema in *Micrurus* envenomation, this effect can worsen the clinical manifestations. However, there are few studies on local inflammatory effects induced by *Micrurus* snake venom. We investigated the edematogenic effect of *Micrurus lemniscatus* venom (MLV) and participation of neuropeptides and mast cells in inflammation. Results demonstrate that MLV induces prominent edema with rapid onset. Using specific pharmacological interferences, we found that MLV-induced edema is dependent on activation of mast cells and substance P-releasing sensory C-fibers. NKA and NKB tachykinins, histamine via H_1_ receptor and serotonin are major mediators of the MLV-induced edematogenic response. These findings suggest that mast cell- and C-fiber-derived mediators are promising therapeutic targets to efficiently counteract the local edema induced by *Micrururs* venoms.

## Introduction

*Micrurus* is one of the four snake genera of medical importance in Brazil. Coral snakes can be found from the southern United States to Argentina [1, 2]. There are at least thirty species in Brazil, and these have a broad geographic distribution and inhabit a variety of habitats [3]. In the state of Bahia, Brazil, *M*. *lemniscatus* is the coral snake responsible for most envenomations, accounting for 0.3% of all accidents caused by snakes every year [4]. Micrurine envenomation is characterized by neurotoxic symptoms, including palpebral ptosis followed by ophthalmoplegia, dysarthria, and dysphagia, and may lead to dyspnea and death as a result of muscle paralysis and respiratory arrest [5–7]. Some reports have shown that, in addition to its neurotoxic action, *Micrurus* venom exhibits myotoxic [8, 9], hemorrhagic [9, 10], hemolytic [11, 12] and edematogenic activities [11, 13]. *Micrurus lemniscatus* venom (MLV) has been reported to have myotoxic [8, 9] and neurotoxic activities in avian and mammalian isolated neuromuscular preparations and to act preferentially on postsynaptic nicotinic receptors without affecting adjacent muscle membranes [11]. It has also been shown to exhibit edematogenic and phospholipase A_2_ activities [9, 14, 15] and to activate the complement system by the lectin pathway [16]. In this context, we have recently shown that a phospholipase A_2_ isolated from MLV exhibits edematogenic activity [17]. However, as the species comprises a complex with many subspecies and a wide geographic distribution, manifesting a variety of different biological activities, and as the neurogenic mechanisms involved in MLV-induced edema have not yet been investigated, further studies of the whole venom are required.

Neurogenic inflammation is a local inflammatory response triggered by the release of neuropeptides (tachykinins), especially substance P (SP) and calcitonin gene-related peptide (CGRP), from sensory nerves (C-fiber neurons) and activated inflammatory cells, particularly mast cells (MCs) [18,19]. MCs are derived from hematopoietic progenitors (myeloid cells) and complete their maturation in peripheral tissues, including the skin, gastrointestinal tract, and airways, where they are in close contact with the outside environment. Because they are found at the interface between the host and the external environment, MCs are considered first-line defenders against invading pathogens [20]. They release numerous vasoactive and proinflammatory mediators, including preformed molecules stored in secretory granules (histamine, serotonin, proteases and tumor necrosis factor α –TNF-α), and release newly synthesized leukotrienes, prostaglandins and platelet-activating factor, as well as many cytokines and chemokines [21].

While viperid venoms are known to trigger prominent localized inflammation and some of these venoms have been associated with activation of afferent fibers and mast cells [22–24], there is no information about the contribution of neuropeptides and mast cells to the local inflammatory response elicited by elapid venoms. This study therefore sought to investigate to what extent (1) MLV can induce local edema and (2) whether neurogenic mediators and mast cells participate in this inflammatory effect.

## Methods

### Animals

Male Wistar rats (160–180 g) were housed in conventional cages in a temperature-controlled room at 23–25°C with a 12 h light/dark cycle. They received standard diet and water *ad libitum* until use.

### Ethics statement

Experiments were approved by the Experimental Animals Committee of the UFBA Institute of Health Sciences (CEUA-ICS) (reference number 091/2015) and complied with recommendations of the Brazilian National Council for the Control of Animal Experiments (CONCEA) in accordance with procedures established by the University Federation for Animal Welfare.

### Venom and drugs

Crude *Micrurus lemniscatus* venom was obtained by manual extraction from specimens captured in the state of Bahia, Brazil (South central region, North central region and Metropolitan region of Salvador) and kept in the Núcleo Regional de Ofiologia e Animais Peçonhentos da Bahia (NOAP), Federal University of Bahia, which is authorized to collect and maintain snakes for scientific research (Instituto Brasileiro do Meio Ambiente e Recursos Naturais Renováveis–IBAMA license no. 016/2002). The vacuum-dried venom was stored at 4°C until use. Thioperamide and methysergide were purchased from Research Biochemical International (RBI, EUA). Promethazine, compound 48/80 (C48/80), capsaicin, HOE 140 and substance P were purchased from Sigma-Aldrich, Brazil. SR 140333 and SR 48968 (Sanofi Recherche, Montpellier, France) were kindly provided by Dr. Soraia Costa (Instituto de Ciências Biomédicas, Universidade de São Paulo, SP Brazil).

### Evaluation of paw edema

MLV was dissolved in 0.9% (w/v) apyrogenic saline, and 0.1 mL of final solutions containing 1 to 10μg/100 μL were injected into the subplantar surface (i.pl.) of the right hind paw of the rats. The contralateral paw received an equal volume of sterile saline without MLV by the same route as a negative control. Prior to injection, the venom solution was filtered through a 0.22 mm Millipore filter (Millipore Ind. Com. Ltda., Brazil). Volumes of both hind paws were measured using a plethysmometer before and at various time points (5 min, 15 min, 30 min, 1 h, 3 h, 6 h and 24 h) after i.pl. injection of MLV according to the method of Van Arman et al. [25]. Results were calculated as the difference between hind paws and expressed as the percentage increase in paw volume.

### Histological assessment of mesenteric mast cell degranulation

To investigate mesenteric mast-cell degranulation by MLV, rats were injected intraperitoneally (i.p.) with doses of 1.4 or 2.8 μg MLV/g. Control animals received sterile saline alone. After 15 min the animals were killed by exsanguination under halothane anaesthesia. For histological assessment of mast-cell degranulation, the abdomen was opened and part of the mesentery carefully removed, stained in toluidine blue-formaldehyde solution for 15 min and mounted on a glass slide, with care being taken not to fold or stretch the tissue. Mast-cell degranulation was expressed as the percentage (%) of mast cells with extruded granules, relative to the total mast cells present in the stained mesentery. At least 100 cells were counted per stained tissue [26].

### Pharmacological treatments

Participation of MCs in MLV-induced edema was investigated by treating rats with C48/80, an MC activator, in a well-characterized protocol for depleting MC granules [27, 28]. Rats were treated with increasing doses (0.1 to 5.0 mg/mL) of C48/80 administered i.p. twice a day for five consecutive days before i.pl. injection of venom. Control animals were treated with saline using the same protocol.

To investigate involvement of NK receptors in MLV-induced paw edema, SR 140333 (an NK_1_-receptor antagonist) or SR 48968 (a NK_2_-receptor antagonist) were co-injected i.pl. (1 nmol/paw and 10 nmol/paw, respectively) with venom into the right hind paw [29, 30]. Control animals received MLV co-injected with sterile saline. To deplete substance P from capsaicin-sensitive primary afferent neurons, rats were also treated with capsaicin (15, 30 and 50 mg/kg) subcutaneously (s.c.) for four consecutive days) [31, 32]. To ascertain involvement of bradykinin, MLV was co-injected with HOE 140, a BK_2_-receptor antagonist, into the hind paw (5 μg/paw, i.pl.) [33]. To evaluate participation of biogenic amines, promethazine, a histamine type 1 receptor (H_1_R) antagonist (5 mg/kg, i.p.) or thioperamide, a histamine type 3 and 4 receptor (H_3_R/H_4_R) antagonist (5 mg/kg, i.p.) or methysergide, a nonselective 5-HT receptor antagonist (5 mg/kg, i.p.) was injected 30 min before administration of venom. Doses of the drugs used were chosen based on published reports [34, 35].

### Statistical analysis

Results are expressed as means ± SEM. Differences between groups were analyzed by analysis of variance (ANOVA) followed by Tukey’s test. Differences with an associated probability (*p* value) of *p* < 0.05 were considered significant.

## Results

### Intraplantar injection of MLV induces edema formation

**Fig 1:**
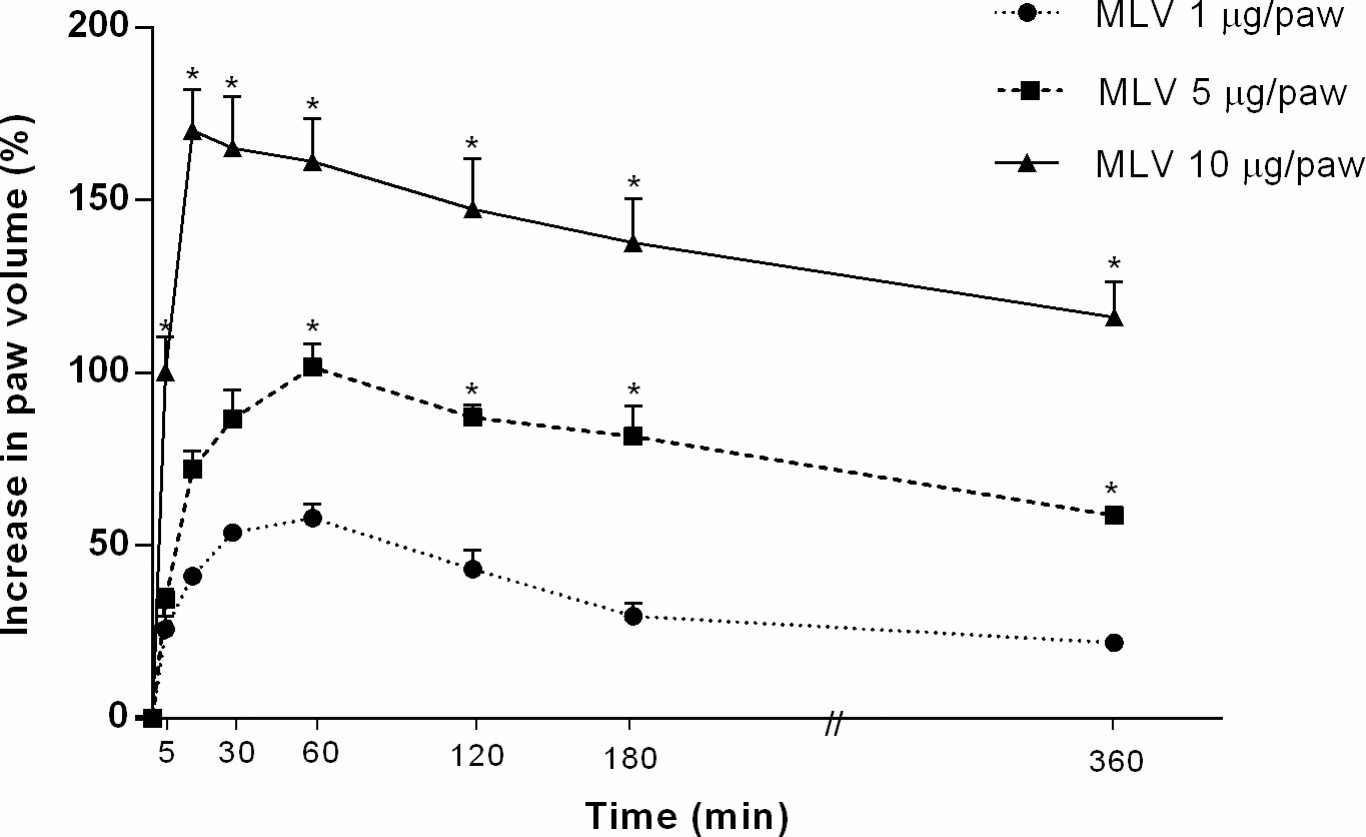
Time course of rat paw edema induced by selected doses of *Micrurus lemniscatus* venom. Increased paw volume was determined at various time points after intraplantar injection of MLV (1–10 μg/paw) into one paw and apyrogenic saline into the contralateral paw (control paw). The increase in volume of each paw (edema) was measured using a plethysmometer. Differences between paws were expressed as a % increase in paw volume. Values are the mean ± SEM of five animals. * *p* < 0.05 compared with MLV (1 μg/paw).

Intraplantar injection of MLV (1–10 μg/paw) into the right hind footpad of the rats caused a time-dependent, rapid-onset edema that peaked between 15 min and 1 h, with an increase in volume of more than 80%, 15 and 30 min after injection. The increase in volume with 10 μg/paw exceeded 160% and remained high until 3 h post-injection, decreasing gradually over the next 6 h and disappearing within 24 h (Fig 1). Based on these results, all experiments involving inflammatory mediators in edema were performed with a 5 μg/paw dose.

### Mast cells are involved in MLV- induced edema

**Fig 2:**
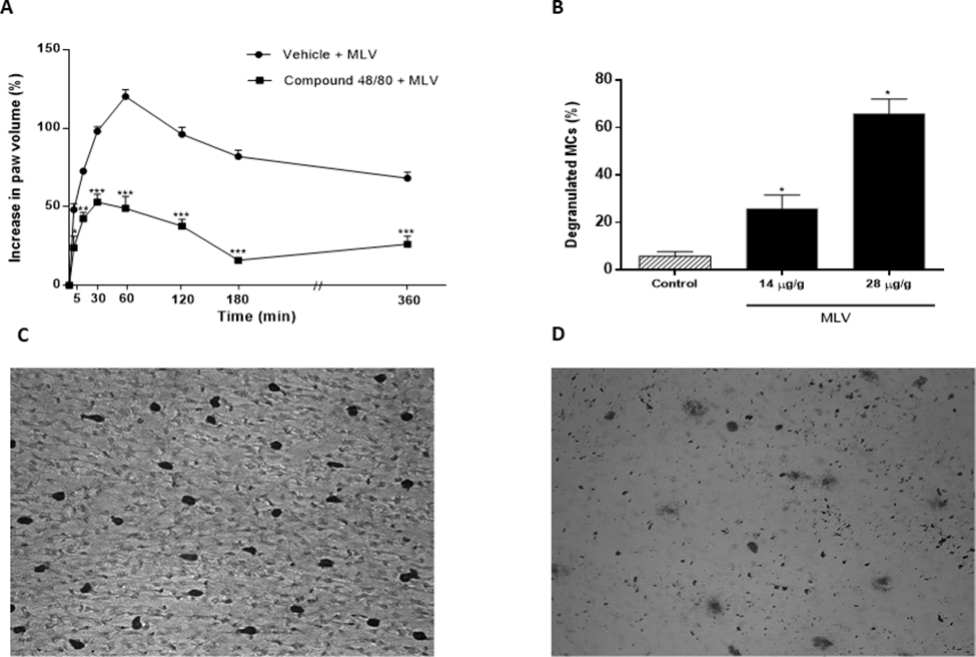
Participation of mast cells (MCs) in paw edema induced by *Micrurus lemniscatus* venom. (A) Groups of rats were treated with compound 48/80 (0.1 to 5.0 mg/mL i.p. twice a day for 5 consecutive days) or an equal volume of vehicle i.p. (control) before MLV injection (5 μg/paw). Edema was evaluated using a plethysmometer at the time points shown. Data are expressed as % increase in paw volume compared with the control paw. (B) Degranulation of mesenteric mast cells was assessed 15 min after venom injection by counting the percentage of cells with extruded granules in the histological preparation. (C, D) Toluidine blue staining of mesenteric mast cells treated with apyrogenic saline (C) or MLV (2.8 μg/g) (D). The mesentery had minimal mast cell degranulation after i.p. injection with apyrogenic saline and extensive mast cell degranulation after injection with MLV. Values are the mean ± SEM of five animals. * *p* < 0.05 compared with control group.

Depletion of mast cells with C48/80 led to a significant reduction in MLV-induced hind-paw edema compared with respective controls (Fig 2A). This effect was observed from 5 min to 3 h post-injection. Injection of MLV into the peritoneal cavity induced significant degranulation of mesentery mast cells at doses of 250 and 500 μg/animal (30% and 70%, respectively) (Fig 2B–2D). Degranulation in negative controls was less than 10%.

### Vasoactive amines participate in MLV- induced edema

**Fig 3:**
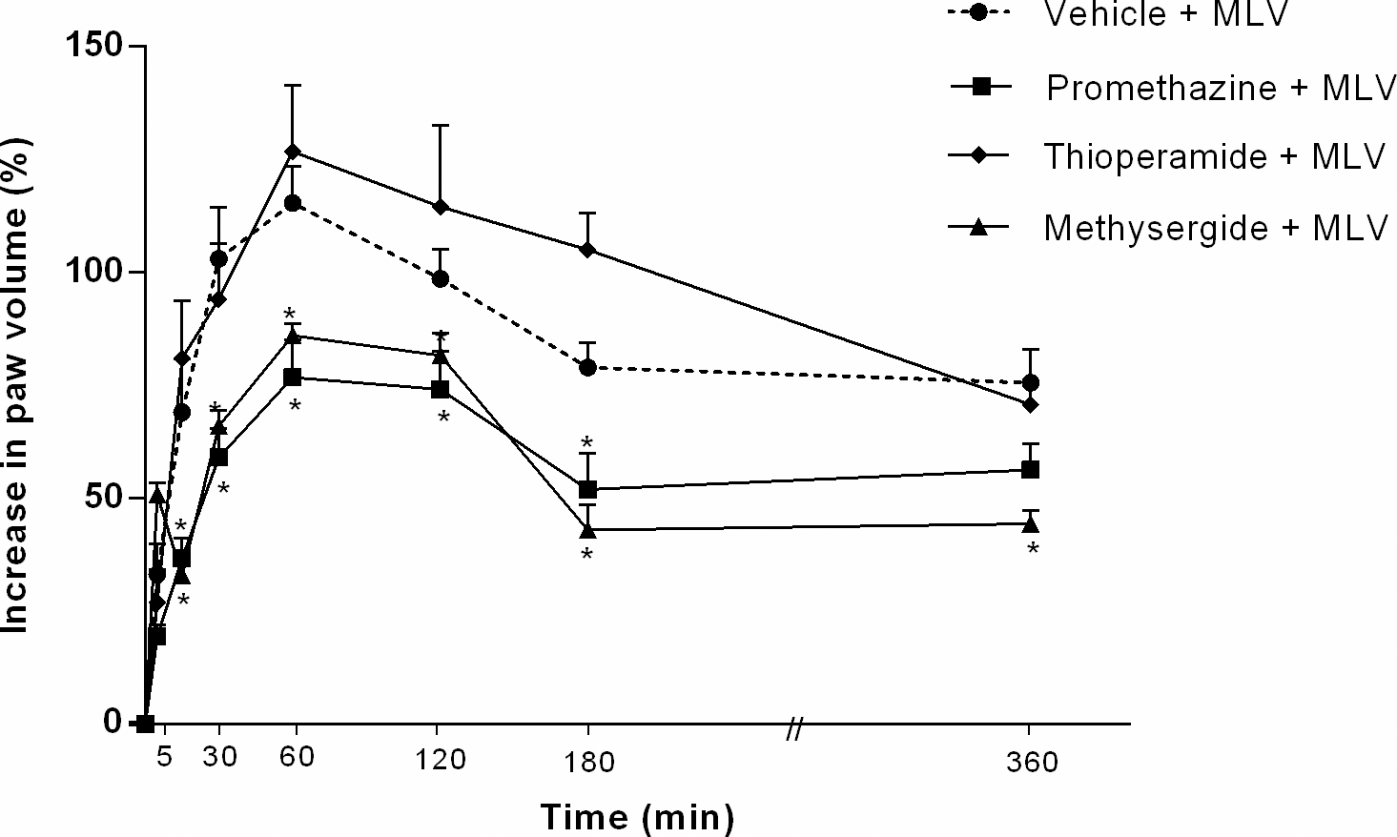
Effect of biogenic amine-receptor antagonists on paw edema induced by *Micrurus lemniscatus* venom. Groups of animals were treated with promethazine (5 mg/kg, i.p.) (an H_1_ receptor antagonist), thioperamide (5 mg/kg, i.p.) (an H_3_R/H_4_R receptor antagonist) or methysergide (5 mg/kg, i.p.) (a nonselective 5-HT receptor antagonist) 30 min before intraplantar injection of MLV (5 μg/paw). The increase in volume of each paw (edema) was measured using a plethysmometer. Data were calculated as the difference between both paws and are expressed as a % increase in paw volume. Values are the mean ± SEM of five animals. * *p* < 0.05 compared with control group.

To investigate the role of mast cell-derived amines, animals were pretreated with promethazine (5 mg/kg, i.p.) and methysergide (5 mg/kg, i.p.) 30 min before injection of MLV (5 μg/paw, i.pl.). Both treatments markedly reduced MLV-induced edema formation until the 3 h post-injection (36.5 ± 2.8% and 33.6 ± 5.4% reduction, respectively). Pre-treatment with thioperamide (5 mg/kg, i.p.) treatment did not affect MLV-induced edema in comparison with control animals (Fig 3).

### Sensory neurons and tachykinins participate in MLV- induced edema

**Fig 4:**
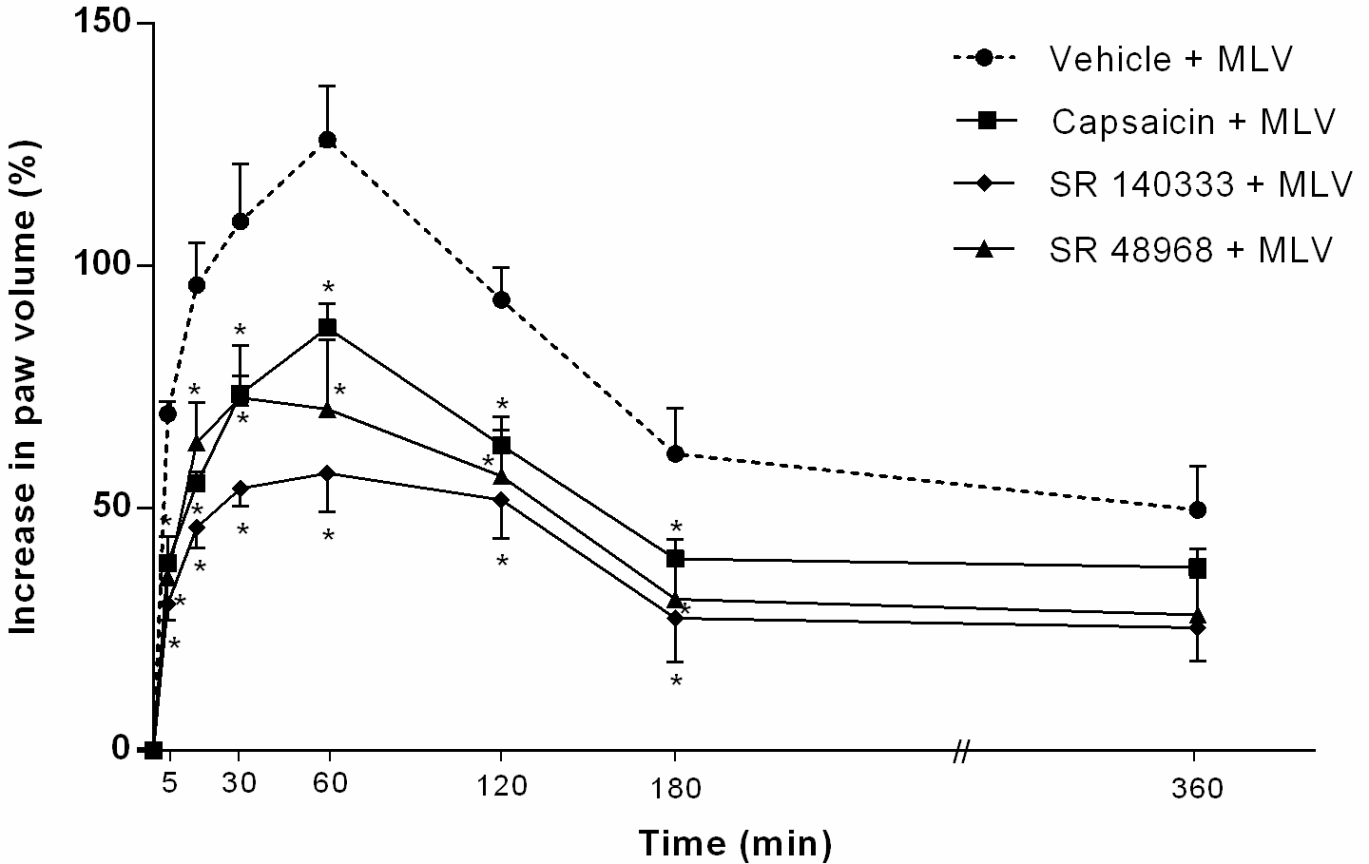
Effect of capsaicin and tachykinin NK_1_- and NK_2_-receptor antagonists (SR14033 and SR 48968, respectively) on edema induced by *Micrurus lemniscatus* venom. Groups of animals were treated with capsaicin (15, 30 and 50 mg/kg, s.c.) for 4 consecutive days to deplete substance P from sensitive primary afferent neurons or NK_1_- or NK_2_ receptor antagonists. Both SR 140333 (1 nmol/paw) and SR 48968 (10 nmol/paw) were co-injected i.pl. with MLV (5 μg/paw). The increase in volume of each paw (edema) was measured using a plethysmometer. Results were calculated as the difference between hind paws and are expressed as a % increase in paw volume. Values are the mean ± SEM of five animals. * p < 0.05 compared with control group.

MLV-induced paw edema was reduced by 56.3% and 49.5%, respectively, by co-injection of venom with tachykinin NK_1_- and NK_2_-receptor antagonists. Treatment with SR 140333, a highly selective non-peptide NK_1_-receptor antagonist ((S)1-(2-[3-(3,4-dichlorophenyl)-1-(3-isopropoxyphenylacetyl)piperidin-3-yl]ethyl)-4-phenyl-1-azoniabicyclo[2.2.2]octane chloride) [36, 37] significantly decreased MLV-induced paw edema in comparison with controls between 15 min and 3 h after injection. Co-injection of SR 48968 ((S)-N-methyl-N[4-(4-acetylamino-4-phenylpiperidino)-2-(3,4-dichlorophenyl)butyl]benzamide), a non-peptide NK_2_-receptor antagonist, also significantly decreased MLV-induced edema. There was no statistically significant difference in the reduction in edema caused by these receptor antagonists (Fig 4). To confirm the participation of tachykinins in MLV-induced edema, animals were treated with capsaicin (15, 30 and 50 mg/kg, s.c., for four consecutive days). While there was significant inhibition of paw edema from 15 to 180 min compared with controls, the reduction was less than that produced by the above receptor antagonists (Fig 4).

### Bradykinin does not contribute to MLV- induced edema

**Fig 5:**
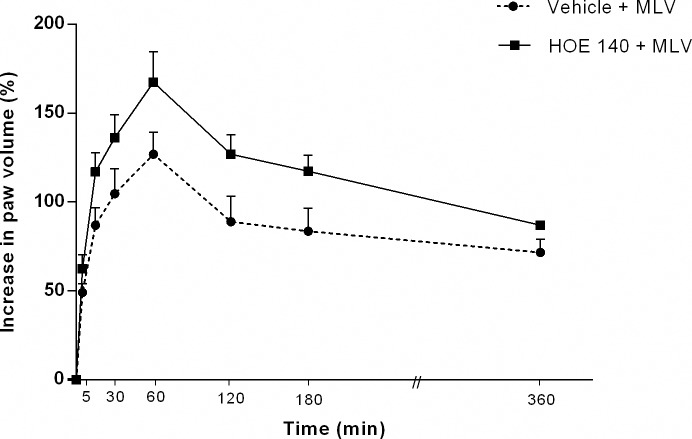
Effect of bradykinin BK_2_-receptor antagonist (HOE 140) on paw edema induced by *Micrurus lemniscatus* venom. Groups of animals were treated with HOE 140 (5 μg/paw) concomitantly with MLV (5 μg/paw, i.pl.). The increase in volume of each paw (edema) was measured using a plethysmometer. Results were calculated as the difference between hind paws and are expressed as a % increase in paw volume. Values are the mean ± SEM of five animals. None of the results were statistically significant.

Treatment of animals with HOE 140, a BK_2_ receptor antagonist, (5 μg/paw, co-injected with venom) did not significantly alter MLV-induced edema compared with controls (Fig 5).

## Discussion

The present results indicate that MLV is capable of inducing edema at the injection site. This effect is dose-dependent and characterized by rapid onset with a peak 1 h after administration, followed by a gradual decline over the following 6 h. These data are consistent with those of previous studies showing that *Micrurus* venoms induce increased vascular permeability at the injection site [38], a phenomenon required for microvascular leakage, with plasma extravasation and edema formation. Our findings are also in agreement with an earlier study that indicated that MLV has inflammatory activities and that these are the result of activation of the complement system [16].

Here, we analyzed participation of selected mediators and inflammatory pathways in MLV-induced paw edema using specific pharmacologic modulation. We found that this MLV-induced effect is dependent on sensory C-fibers, as the edema was significantly reduced by pretreatment of animals with capsaicin, which is widely used to identify sensory neural pathways and to explore their contribution to inflammatory responses. The protocol used here for the daily capsaicin pretreatment causes degeneration of a large percentage of peripheral unmyelinated fibers in rats (dorsal root ganglion neurons) [32, 39]. Our results with capsaicin treatment therefore indicate that MLV-induced edema requires activation of microvascular sensory C-fibers.

Sensory C-fibers are essential components of the nonadrenergic, noncholinergic (NANC) system and are found around blood vessels and mucosal glands within and beneath the epithelium [36, 40]. Activation of peripheral C-fibers by electrical or chemical (capsaicin) stimulus causes the release of neuropeptides known as tachykinins and initiates the cascade of neurogenic inflammation, which plays a major role in the response to tissue injury [36, 41–43]. Once released, the tachykinins trigger tissue-specific responses, such as increased vascular permeability and, consequently, edema formation [42, 44]. They mediate edema formation via activation of three subtypes of receptors known as NK_1_, NK_2_ and NK_3_ with different orders of potency. Substance P, an NK1-receptor agonist, is believed to play a greater role in neurogenic-induced edema than the other tachykinins [43, 45, 46]. Thus, it is likely that MLV stimulates sensory neurons to release tachykinins. Whether MLV exerts a direct or indirect effect on C-fibers was not investigated, but warrants further investigation. In light of the above, we used selective tachykinin NK_1_- and NK_2_-receptor antagonists (SR 140333 and SR 48968) to investigate the contribution of endogenous tachykinins to MLV-induced edema. Our finding that NK_1_-receptor antagonist markedly reduced MLV-induced edema reinforces our observation that sensory nerves are activated by this venom and indicates that neurogenic mediators, particularly substance P, are involved in this edema of neurogenic origin. Furthermore, our results demonstrate that MLV-induced edema was partially reduced by the NK_2_-receptor antagonist, strongly suggesting that in addition to substance P, neurokinin A and/or calcitonin gene-related peptide are released from sensory C-fibers, contributing to the local edema induced by MLV. Taken together, these findings suggest that neurogenic inflammation accounts for in the local edematogenic effect of MLV. While neurogenic inflammation induced by wasp and bee venom [47, 48] and venoms of the spider *Phoneutria nigriventer* [49] and snake *Crotalus durissus* sp. [22] has previously been reported, this is the first demonstration of a neurogenic mechanism in local inflammation induced by *Micrurus* venoms. Corroborating our findings, participation of neurogenic factors in the local hemorrhage induced by *Bothrops jararaca* snake venom has also been previously reported [50].

Plasma extravasation and edema induced by substance P results from activation of endothelial NK_1_ receptors in postcapillary venules and mast cells [19]. Activation of mast cells and the consequent release of inflammatory mediators, including histamine and serotonin, constitute an intermediate step in sensory nerve-mediated responses. Histamine and serotonin act as key mediators of the early phase of inflammation by inducing an increase in vascular permeability, leading to edema formation. Moreover, it has been demonstrated that histamine evokes the release of substance P and calcitonin gene-related peptide, forming a bidirectional link between histamine and neuropeptides and further amplifying neurogenic inflammation [19, 51]. To better understand neurogenic mechanisms triggered by MLV that lead to edema, the effect of this venom was investigated in mast-cell-depleted animals. The finding that depletion of mast cells by C48/80 markedly reduced MLV-induced paw edema indicates that mast-cell-derived mediators contribute to the inflammatory activity of MLV. Supporting this hypothesis, our results revealed a significant reduction in MLV-induced edema following treatment with promethazine or methysergide, indicating that histamine and serotonin, respectively, are involved in this venom-induced effect. Furthermore, our data showing that MLV can induce degranulation of mast cells lend support to the above findings and suggest that release of vasoactive amines from mast cells can be attributed at least partially to the direct action of MLV on this cell population. However, an indirect effect of MLV on mast cells via secondary degranulating agents cannot be ruled out since there are reports that substance P can induce in vivo and in vitro mast cell degranulation, resulting in the local release of vasoactive amines [42, 52, 53]. While venoms of various genera and families have been reported to degranulate mast cells [22–24, 54, 55], this is the first time that mast cells have been shown to be targets of *Micrurus* sp venom.

Even though several studies have shown that bradykinin, an inflammatory mediator that increases vascular permeability and hyperalgesia [56, 57], can stimulate sensory neurons, causing them to release neuropeptides [56–59], our results show that HOE 140, a potent bradykinin BK_2_-receptor antagonist, was ineffective in modifying the effect of MLV, suggesting that bradykinin via BK_2_ receptor is not involved in MLV-induced edema. Consistent with our findings, bradykinin does not seem to be involved in local edema induced by *Bothrops asper* [33] and *Bothrops jararaca* venoms [60] via the BK_2_ receptor, but it has been implicated in local edema induced by *Bothrops lanceolatus* venom in rats [61].

In conclusion, MLV can induce paw edema in rats by mechanisms involving activation of mast cells and local sensory C-fibers. Our results show that tachykinins NKA and NKB, histamine and serotonin are major mediators of the MLV-induced edematogenic response. These mediators may interact with each other or may be released sequentially. Mast cell- and C-fiber-derived mediators should be considered as potential therapeutic targets to interrupt development of local edema induced by *Micrurus* venoms.
